# Analysis of the Paired TCR α- and β-chains of Single Human T Cells

**DOI:** 10.1371/journal.pone.0037338

**Published:** 2012-05-23

**Authors:** Song-Min Kim, Latika Bhonsle, Petra Besgen, Jens Nickel, Anna Backes, Kathrin Held, Sigrid Vollmer, Klaus Dornmair, Joerg C. Prinz

**Affiliations:** 1 Department of Dermatology, Ludwig-Maximilian-University, Munich, Germany; 2 Institute of Clinical Neuroimmunology, Ludwig-Maximilian-University, Munich, Germany; 3 Max-Planck-Institute of Neurobiology, Martinsried, Germany; University of Muenster, Germany

## Abstract

Analysis of the paired i.e. matching TCR α- and β-chain rearrangements of single human T cells is required for a precise investigation of clonal diversity, tissue distribution and specificity of protective and pathologic T-cell mediated immune responses. Here we describe a multiplex RT-PCR based technology, which for the first time allows for an unbiased analysis of the complete sequences of both α- and β-chains of TCR from single T cells. We validated our technology by the analysis of the pathologic T-cell infiltrates from tissue lesions of two T-cell mediated autoimmune diseases, psoriasis vulgaris (PV) and multiple sclerosis (MS). In both disorders we could detect various T cell clones as defined by multiple T cells with identical α- and β-chain rearrangements distributed across the tissue lesions. In PV, single cell TCR analysis of lesional T cells identified clonal CD8^+^ T cell expansions that predominated in the epidermis of psoriatic plaques. An MS brain lesion contained two dominant CD8^+^ T-cell clones that extended over the white and grey matter and meninges. In both diseases several clonally expanded T cells carried dual TCRs composed of one Vβ and two different Vα-chain rearrangements. These results show that our technology is an efficient instrument to analyse αβ-T cell responses with single cell resolution in man. It should facilitate essential new insights into the mechanisms of protective and pathologic immunity in many human T-cell mediated conditions and allow for resurrecting functional TCRs from any αβ-T cell of choice that can be used for investigating their specificity.

## Introduction

T cells are essential for protective immunity against infections and malignant tumors. Furthermore, they may confer pathogenic immune responses in autoimmune disorders such as multiple sclerosis, psoriasis vulgaris, rheumatoid arthritis, type I diabetes, inflammatory bowel disease and others. Advances in understanding the mechanisms of protective and pathogenic T-cell responses in man have been hampered by the fact that often neither the relevant T cell populations nor their antigen specificity could be defined. T-cell receptor (TCR) analysis has been used to address these issues but provided only incomplete insights. Most techniques to analyze the human TCR repertoire were limited to TCR β-chains, i.e., they revealed only “half the truth”, while an unbiased characterization of complete TCR molecules could not yet be achieved [1]. Monoclonal TCR antibodies are only available for a limited number of TRBV regions. RT-PCR-amplification, spectratyping, and random cloning of TCR β-chain transcripts provided only estimates of the heterogeneity of T-cell populations. A technique to analyse α-chains from single T cells obtained by laser-microdissection was limited to clonally expanded T cell populations that could be identified by anti-TCR Vβ-chain antibodies [2]. An unbiased characterization of the paired, i.e. complementary TCR α- and TCR β-chain from single human T cells, which would be required to define the specificity of T-cell responses [3], however, failed hitherto due to the huge diversity of genetic elements from which functional TCR αβ transcripts are generated [4]. As a consequence, the pathomechanism of many human T-cell mediated conditions have remained elusive.

To overcome this obstacle in the characterization of T-cell mediated immunity we developed a multiplex RT-PCR-based protocol to determine paired αβ-TCR rearrangements from any single T cell of choice without prior knowledge of their particular TRAV or TRBV usage. We assessed its capacity to characterize pathologic T-cell infiltrates in two T cell-mediated autoimmune disorders, psoriasis vulgaris (PV) and multiple sclerosis (MS).

PV is a chronic, recurrent inflammatory skin disease resulting from a T-cell mediated immune response in the skin [5]. Former analyses of TCR β-chain repertoires of lesional psoriatic T-cell infiltrates by random amplification, cloning and sequencing of TCR β-chain cDNA from lesional biopsies had suggested select oligoclonal T-cell expansions [6], [7], [8], [9], but the precise clonality, subtype and tissue distribution of the pathogenic T cells has remained unknown. MS is believed to be an autoimmune disease where clonally expanded lymphocytes can be detected in different regions of the brain [10], [11] and CD8^+^ T cells are thought to mediate an inflammatory attack on the central nervous system [12], [13]. Expanded CD8^+^ T cell clones were shown to dominate the lesion-infiltrating immune cell population [10]. However, in these studies only TCR β-chain rearrangements were analysed, while the matching TCR α-chains could not be determined.

In both disorders we could identify T-cell clones, characterize their matching TCR α- and β-chain rearrangements and show their tissue distribution on the single cell level. Accordingly, our technology should have wide applications and promote research into the nature and antigen-specificity of many medically important protective or pathologic T-cell responses, allowing for the development of novel diagnostic, therapeutic or preventive strategies.

## Results

### Strategy and Primer Design

We established an RT-PCR-based method for the molecular analysis of the matching i.e. paired αβ-TCR rearrangements of single human T cells. For this purpose we developed nine degenerate PCR primers (termed Vp1 to Vp9, Table 1), which cover the entire repertoire of functional TCR β-gene V-elements and, together with 24 TCR Vα-*out* primers for the Vα repertoire developed recently [2], can simultaneously amplify the cDNA of all functional human TCR Vα- and Vβ-region gene transcripts by multiplex RT-PCR.

**Table 1:** Sequence, localisation and specificity of oligonucleotide primers.

Primer	Vβ specificity*	Nucleotide sequence	Localisation
**Vp1**	1, 5, 16, 17, 23	5′-T**S**Y TTT GTC TCC TGG GAG CA -3′	Leader segm.
**Vp2**	22, 25	5′-CCT GAA GTC GCC CAG ACT CC -3′	Vβ gene
**Vp3**	18, 24	5′-GTC ATS CAG AAC CCA AGA **Y**AC C -3′	Vβ gene
**Vp4**	2, 4	5′-GG**W** TAT CTG T**M**A G**M**G TGG AAC CTC -3′	Vβ gene
**Vp5**	3, 11, 12,	5′-ATG TAC TGG TAT CGA CAA GA**Y** C -3′	Vβ gene
	13, 14, 15		
**Vp6**	20	5′-CAC TGT GGA AGG AAC ATC AAA CC -3′	Vβ gene
**Vp7**	6, 8, 21	5′-TCT CCA CTC T**S**A AGA TCC AGC -3′	Vβ gene
**Vp8**	6	5′-CAG **R**AT GTA **R**AT YTC AGG TGT GAT CC -3′	Vβ gene
**Vp9**	7, 9	5′-CCA GAC **W**CC AAR A**Y**A CCT GGT CA -3′	Vβ gene
**UP**		5′-ACA GCA CGA CTT CCA AGA CTC A -3′	
**Vp2-UP**		5′-ACA GCA CGA CTT CCA AGA CTC A CCT GAT GTC	
		GC**C** CAG ACT CC -3′	
**Vp9-UP**		5′-ACA GCA CGA CTT CCA AGA CTC A TCA GAC WCC	
		AAR AYA CCT GGT CA -3′	
**Cβ-** ***out***	Cβ	5′-TGG TCG GGG AAG AAG CCT GTG -3′	Cβ gene
**Cβ -** ***in***	Cβ	5′-TCT GAT GGC TCA AAC ACA GC -3′	Cβ gene
**Cα-** ***out***	Cα	5′-GCA GAC AGA CTT GTC ACT GG -3′	Cβ gene
**Cα-** ***in***	Cα	5′ -AG TCT CTC AGC TGG TAC ACG -3′	Cβ gene

* Arden nomenclature [27]; degenerate primers contain bold letters to indicate nucleotide exchanges. All primers Vp1 to Vp9 were also synthesised with a “UP” sequence at their 5′-end (termed Vp1-UP to Vp9-UP). We only show Vp2-UP and Vp9-UP because nucleotides exchanges (underlined) were introduced there to avoid primer interactions.

The Vβ-primers were designed as degenerate primers utilizing sequence homologies of the various Vβ-gene families. To maintain a high specificity we allowed only one mismatch with the primary nucleotide Vβ-gene sequence. Except Vp1, which is located in the leader segment, all primers are positioned within the Vβ genes. Primer interactions with the Vα-primer set were excluded first by *in silico* analysis using several software programs and then experimentally by testing numerous primer combinations by RT-PCRs on mRNA from peripheral blood T cells.

**Figure 1:**
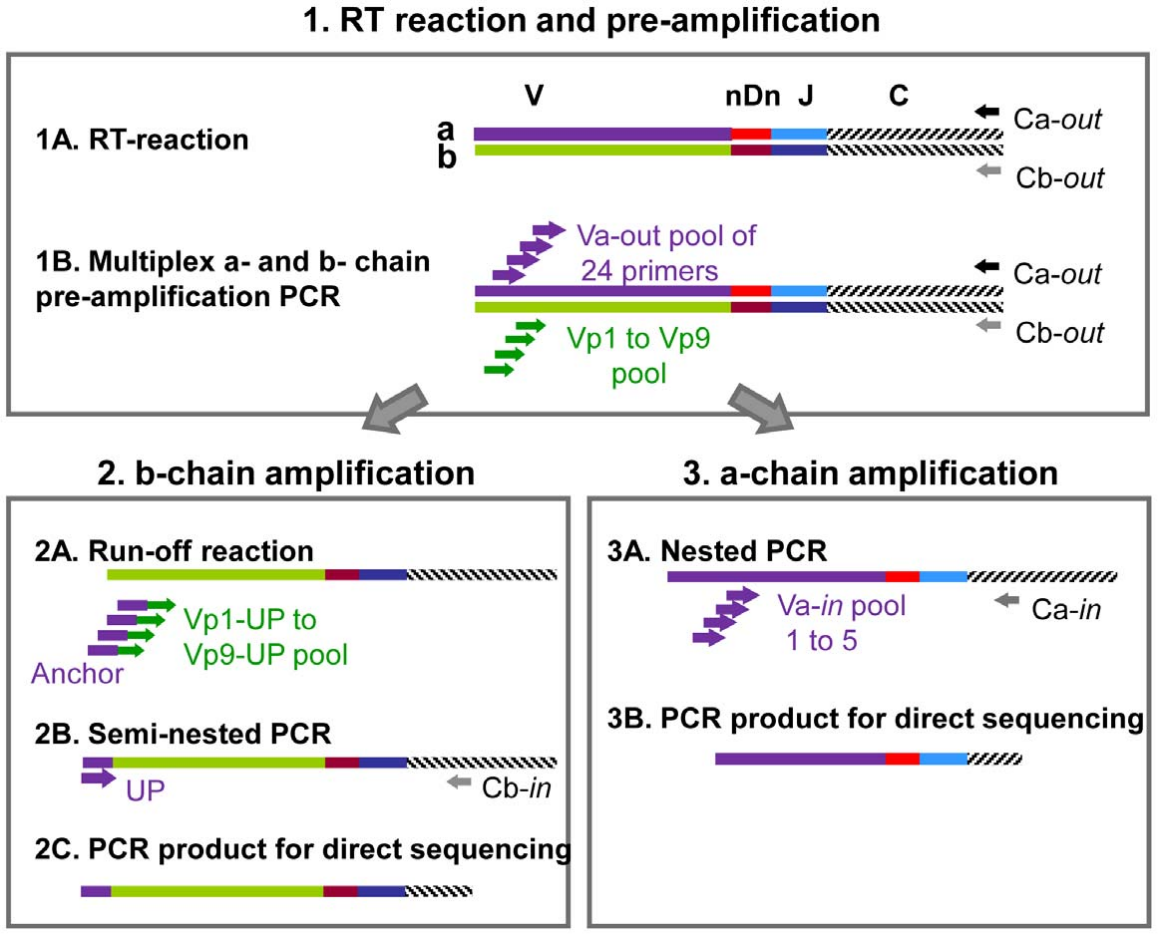
Strategy to identify paired TCR α- and β-chains from single T cells. **Step 1**: one step multiplex pre-amplification RT-PCR composed of step 1A, reverse transcription using a Cα-*out* and Cβ-*out* primer and step 1B, amplification of the α and β TCR rearrangements by a pool of 9 oligonucleotide Vβ primers (Vp1– Vp9) for the Vβ repertoire and a pool of 24 oligonucleotide Vα primers for the Vα repertoire. Subsequently, the PCR products of the Vβ (step 2) and Vα (step 3) gene rearrangements are handled separately. **Step 2**: Introduction of a universal primer (UP) site at the 5′-site of each Vβ-rearrangement using primers Vp1-UP to Vp9-UP by a run off reaction (step 2A), followed by amplification of the Vβ gene rearrangement by seminested PCR with the UP primer and a Cβ-*in* primer (step 2B) and sequencing (step 2C). **Step 3**: Amplification (step 3A) and sequencing (step 3B) of the Vα gene rearrangement by nested PCR from the pre-amplification PCR product using five different nested Vα-*in* primer pools and a Cα-*in* primer.

The strategy for the molecular characterization of αβ-TCR rearrangements of single T cells is outlined in Figure 1. It starts with a one step multiplex pre-amplification RT-PCR, where reverse transcription is done by primers specific for the α- and β-constant region genes (step 1A). Then the TCR α- and β-chains are amplified with our novel Vβ-gene specific primer set (Table 1) and the “outer primer” set for the Vα-region genes [2] (step 1b). The pre-amplification PCR products then served as templates to amplify the β-chains by anchor PCR (step 2) and the α-chains (step 3) by nested PCR.

For anchor PCR we introduced a unique 22-mer oligonucleotide as a universal anchor sequence (termed “UP”) to the 5′ end of the nine different Vβ-gene forward primers (Table 1). The pre-amplification RT-PCR product was subjected to a run-off PCR using these nine anchored primers (termed Vp1-UP to Vp9-UP, Figure 1, step 2A). Next we amplified the respective single cell TCR β-chain rearrangement independent from the TCR Vβ-gene family using the “UP” anchor primer together with a nested Cβ-*in* primer in a third PCR (step 2B). When a PCR product for the TCR β-chain was obtained, we amplified the corresponding TCR α-chain rearrangement from the pre-amplification multiplex RT-PCR product in five different nested PCRs (step 3A) using five Vα-*in* primer pools for the Vα repertoire described recently [2]. The amplified TCR α- and β-chain rearrangements were characterized by direct sequencing (steps 2C and 3B).

### Functional Verification of the Primer Sets

**Figure 2:**
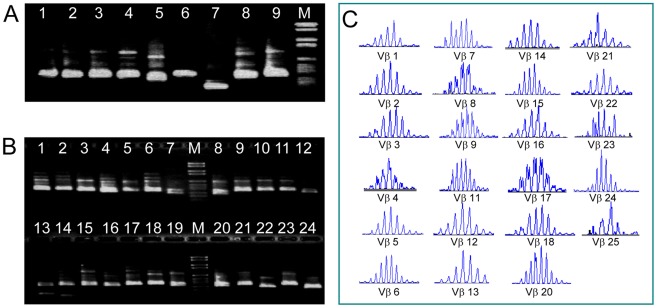
Functional validation of the primer sets for the simultaneous amplification of the TCR α- and β-gene repertoires by multiplex RT-PCR. cDNA from PBL was amplified with 24 Vα and 9 Vβ primers in a multiplex RT-PCR. (**A**) nine forward Vβ-primers (Vp1 to Vp9) were used together with the Cβ-*out* reverse primer (lanes 1–9). (**B**) 24 Vα-*out* forward primers were used together with the reverse Cα-*out* primer (lanes 1–24) in individual reactions (see ref. (2) for α-primer sequences and correlation of the lanes to Vα-families). Each Vβ- (**A**) and Vα-primer (**B**) yielded a PCR product of the expected size. M, molecular weight marker. (**C**) To validate that all TCR Vβ-gene families were covered, the pre-amplification multiplex RT-PCR product was amplified using 23 Vβ-primers specific for the functional Vβ-gene repertoire together with the FAM-labelled Cβ-*in* reverse primer in individual reactions as described [14]. The PCR-products were analyzed by spectratyping their fragment-lengths on a genetic sequencer.

We validated the specificity and compatibility of the 9 Vβ-gene primers and the Cβ-*out* primer in various PCRs together with the Vα primers. Each Vp-primer yielded a PCR product of the expected size. In Figure 2 we show an experiment where we reverse transcribed mRNA of peripheral blood T cells with the Cα-*out* and Cβ-*out* primers and amplified the transcripts with the 24 Vα- and 9 Vβ-primers in the pre-amplification multiplex RT-PCR. We then selectively amplified the rearrangements of the different Vα- and Vβ-gene families in a second (Vβ) and third (Vα) round of nested PCRs using the nine anchor-forward Vβ-primers with the Cβ-*in* reverse primer, or the 24 Vα-*in* forward primers with the reverse Cα-*in* primer [2] in individual reactions. Each Vβ- (Figure 2A, lanes 1–9) and Vα-primer (Figure 2B, lane 1–24) yielded a PCR product of the expected size.

In a second, parallel approach we amplified the pre-amplification multiplex RT-PCR product with 23 Vβ-primers specific for the different functional Vβ-region gene families together with the FAM-labelled Cβ-*in* reverse primer [9]. The fragment-lengths distribution of the hypervariable N(D)N-regions of the PCR products was then determined by PAGE [14]. All amplified TCR Vβ-gene rearrangements revealed spectratypes with Gaussian-like distributions typical of polyclonal T-cell populations in peripheral blood (Figure 2C). Together, these results document that the combined Vα- and Vβ-primer sets were capable of simultaneously amplifying the full functional repertoire of both TCR α- and β-chains.

### Analysis of TCR α- and β-chains of Single T Cells from Peripheral Blood

We tested the efficiency of this approach for single cell TCR analysis using CD4^+^ and CD8^+^ blood T cells. To maintain an optimal viability the T cells had been labelled with magnetic beads and aspirated manually with a pipette in an inverted microscope. In 82 of 96 CD4^+^ (85.4%) and in 76 of 96 (79.2%) CD8^+^ T cells we could amplify and sequence a functional TCR β-chain rearrangement. Among these 158 β-chain rearrangements all functional TCR Vβ-gene families were represented except Vβ25, which is rarely rearranged in general. We did not find a single chain twice, which provides evidence that PCR contaminations can be excluded. We then characterized the TCR α-chain rearrangements exemplarily in 20 CD4^+^ and 20 CD8^+^ T cells where we had identified functional β-chains. We obtained the corresponding TCR α-chains from each individual T cell investigated. Thus, our technique allows the molecular characterization of the paired αβ-TCR rearrangements from single viable T cells with high yields.

When we tested single anti-CD3 labelled T cells from peripheral blood isolated by flow cytometry, the yield for β-chains in 139 analyzed T cells was 27.3% (38/139), and for matching α-chains was 39.5% (15/38). The decreased yields are presumably due to the expected loss and damage of cells during sorting. However, these data show that our method is also suited for high throughput screening experiments.

### TCR Analysis of Single T-cells from the Pathologic Infiltrate in Psoriasis

Next we examined viable, putatively pathogenic T cells from explant cell culture from lesional biopsies of five patients with chronic plaque PV. We divided each biopsy specimen in two parts. From part “A” we isolated single CD4^+^ or CD8^+^ T cells for TCR analysis. To ensure that T cells with identical β-chains belonged to a clonally expanded population, we applied two criteria: Firstly, they had to carry the same Vα rearrangement; Secondly, we tested for select TCR clones whether we could identify the respective β-chain transcript by PCR and direct DNA sequencing in the second part “B” of the biopsy.

In patient #1 we analysed 50 CD4^+^ and in patient #2 50 CD8^+^ T cells. They had been isolated from “full thickness” biopsies consisting of dermis and epidermis. In 40/50 CD4^+^ and 37/50 CD8^+^ T cells functional αβ-TCRs were identified. Three of the 40 CD4^+^ and two of the 37 CD8^+^ T cells had identical TCR Vβ- and Vα-rearrangements (Table 2). Further, we could amplify their TCR β-chain transcripts using clone-specific primers from part “B” of the biopsy. According to the above criteria, these T cells represented clonally expanded populations in the tissue lesion. The other T cells had unique β-chains.

**Table 2:** αβ-TCR rearrangements and frequency of clonal CD4^+^ or CD8^+^ T cells isolated from lesional full thickness skin biopsies of PV patients #1 to #3.

				CDR3β*					CDR3α*						
Patient #	CD Subtset	Identical/analyzed	TCR Vβ-gene	Vβ	NDN	Jβ	Jβ-gene	TCR Vα-gene	Vα	NN	Jα	Jα-gene	Proven in Part B	Proven in PBL	Provenin tonsil
1	4	3/40	6S5	CASS	PTSLT	DTDQ	2.3	12S1	FCA	LSGA	NDYK	20	+	N	N
2	8	2/37	8	CAS	TPSRGIS	YGYT	1.2	18S1	LCA	F	NSGG	13	+	N	N
3	4	2/82	18	CASS	TTPGNS	GNTI	1.3	11S1	CAV	EDGN	TDK	34	+	+	+
	8	2/41	6S6	CASSL	NPS	SGNT	1.3	2S1	CAV	IR	AG	39	+	-	+
	8	3/41	6S5	SSL	SPVAY	SNQP	1.5	1S1	CAV	TD	QAGT	15	+	+	+
								15S1	CAE	P	QGGK	23			
	8	2/41	6S3	CASSL	RPGTGGF	ETQY	2.5	8S1	CAA	SD	SGGG	45	+	+	−
								3S1	FCA	TAPPR	DGQK	16			

* deduced amino acid sequence, one letter amino acid code; +/−, TCR rearrangement also/not identified in cDNA derived from biopsy part A, peripheral blood lymphocytes (PBL), tonsil of patient; N, not available.

In patient #3 we analysed both lesional CD4^+^ and CD8^+^ T cells. This patient had been tonsillectomized due to constant PV flares in association with a recurrent streptococcal angina, which is the main infectious psoriasis trigger [9], [15]. cDNA from blood lymphocytes and fractions of the tonsils were available for analysis as well. 100 CD4^+^ and 50 CD8^+^ T cells were analyzed. In 82 CD4^+^ and 41 CD8^+^ T cells functional αβ TCRs were identified. Two out of the 82 CD4^+^ T cells had identical αβ TCRs. Among the 41 CD8^+^ T cells, we found twice two identical αβ TCRs and once three identical αβ TCRs (Table 2). The respective β-chain rearrangements were also identified within the corresponding part “B” of the biopsy, in peripheral blood and/or the tonsillar tissue of the patient (Table 2). Thus, our technology may identify clonally expanded αβ-T cells in complex T cell populations and track their distribution across different organs.

### Differential Distribution of Psoriatic CD8^+^ T Clones in Epidermis and Dermis

While the majority of the psoriatic T-cell infiltrate is located within the dermis, the development of PV is crucially dependent on the accumulation of CD8^+^ T cells within the epidermal keratinocyte layer [16]. In patients #4 and #5 we therefore separated dermis and epidermis of the lesional biopsies and analysed the differential distribution of CD8^+^ T cells in these skin compartments. In patient #4 (Table 3) 52 of 60 analyzed epidermal and 36 of 45 analyzed dermal T cells yielded functional αβ TCRs. We found nine different αβ-TCR rearrangements in duplicates or triplicates among the 52 epidermal CD8^+^ T cells. Four of these αβ-TCR rearrangements (clones #1, 2, 6, and 9) were also seen in the dermal T cells. The 36 dermal T cells contained only one TCR clone (#10), which was not seen in the epidermis. In patient #5 (Table 4), 33 of 40 analyzed epidermal and 25 of 30 analyzed dermal CD8^+^ T cells yielded functional αβ TCR rearrangements. Three different αβ-TCR rearrangements (clones #1–3) were selectively present in three, four or seven of the 33 epidermal CD8^+^ T cells. Two TCRs (clones #4, 5) were seen in each one epidermal and dermal T cell. Two other T-cell clones (#6 and #7) were only seen in the dermis. For select T-cell clones the Vβ signature was also confirmed in part “B” of the biopsy. Accordingly, our technology of single cell TCR-analysis may define the distribution of particular T-cell clones within heterogeneous T-cell infiltrates. For PV our results indicate that CD8^+^ T-cell clones may predominate within the epidermis.

**Table 3:** αβ-TCR rearrangements, frequency and tissue localization of clonal CD8^+^ T cells isolated from lesional dermis and epidermis of PV patient #4.

				CDR3β					CDR3α				
Clone #	Epidermal T cells identical/analyzed	Dermal T cells identical/analyzed	TCR Vβ-gene	Vβ	NDN	Jβ	Jβ-gene	TCR Vα-gene	Vα	NN	Jα	Jα-gene	Proven in part B
1	2/52	1/36	7S1	CASSQ	ENRG	YEQY	2.7	12S1	YFCA	LSGA	NDYK	20	N
2	3/52‡	1/36	13S1	CASSY	SEGED	EAFF	1.1	3S1	YFCA	TDAL	YSGG	45	+
3	2/52‡	-	1S1	CASS	PRGGE	NTIY	1.3	7S2	YLCA	VL	NDYK	20	+
4	2/52	-	21S3	CASS	STLAGGP	DTQY	2.3	2S1	YLCAV	TP	TDKL	34	N
5	2/52	-	21S3	CASS	LGRL	QETQ	2.5	6S1	YFCA	MRDY	QGGK	23	+
								11S1	YCAV	EDG	NTDK	34	
6	2/52	1/36	21S2	CASS	PAQ	-	-	1S2	YFCAV	SF	YNQG	23	N
7	2/52	-	18	CAS	AGTGYF	QPQH	1.5	3S1	YFCA	TDPD	SGGG	45	N
8	2/52	-	17	CAS	TLRSSG	NEKL	1.4	22S1	YFCA	LISM	DSNY	33	N
9	1/52	1/36	13S3	CAS	TELAGD	YNEQ	2.1	23S1	YLCA	VY	TGGF	9	N
10	-	2/36	6S4	CAS	WTGELG	GYTF	1.2	18S1	YLCA	F	NSGG	13	N
								12S1	YFCA	LSEA	GNTG	37	

See legend to Table 2 for details. **‡**: T cells were in direct contact with antigen-presenting cells of dendritic or epithelial morphology; N, not analyzed.

**Table 4:** αβ-TCR rearrangements, frequency and tissue localization of clonal CD8^+^ T cells isolated from lesional dermis and epidermis of PV patient #5.

				CDR3β					CDR3α				
Clone #	Epidermal T cells identical/analyzed	Dermal T cells identical/analyzed	TCR Vβ-gene	Vβ	NDN	Jβ	Jβ-gene	TCR Vα-gene	Vα	NN	Jα	Jα-gene	Proven in part B
1	3/33	-	21S2	CASS	PRTSGG	YNEQ	2.1	8S1	YFCA	PPR	DTGR	5	N
								15S1	YFCAE	SIK	DTGR	5	N
2	7/33	-	2S1	CSAR	DQGQHR	TDTQ	2.3	5S1	YLCA	SGLA	GNQF	49	+
3	4/33	-	2S1	CSAR	GGLGLMP	GELF	2.2	28S1	AVYLCA	VAT	SGGG	45	+
4	1/33	1/25	7S1	CASSQ	LTSESY	SYNE	2.1	25S1	YFCA	GI	NAGN	38	N
5	1/33	1/25	13S3	CAS	GWDRGT	FFGQ	1.1	2S1	YLCA	V	SGGY	4	N
6	-	2/25	9S1	CASSQ	DLWTGGWG	TDTQ	2.3	2S1	YLCAV	VSA	NSGG	53	N
7	-	2/25	8S1	CASSL	ILGGD	EQYF	2.7	12S1	VYFCA	LRGY	NTNAG	27	N

See legend to Tables 2 and 3 for details.

A total of 425 T cells isolated by manual aspiration from the PV biopsy specimens was analyzed by single cell TCR analysis. The overall yield was 346 functional αβ TCR rearrangements (81.4%).

**Figure 3:**
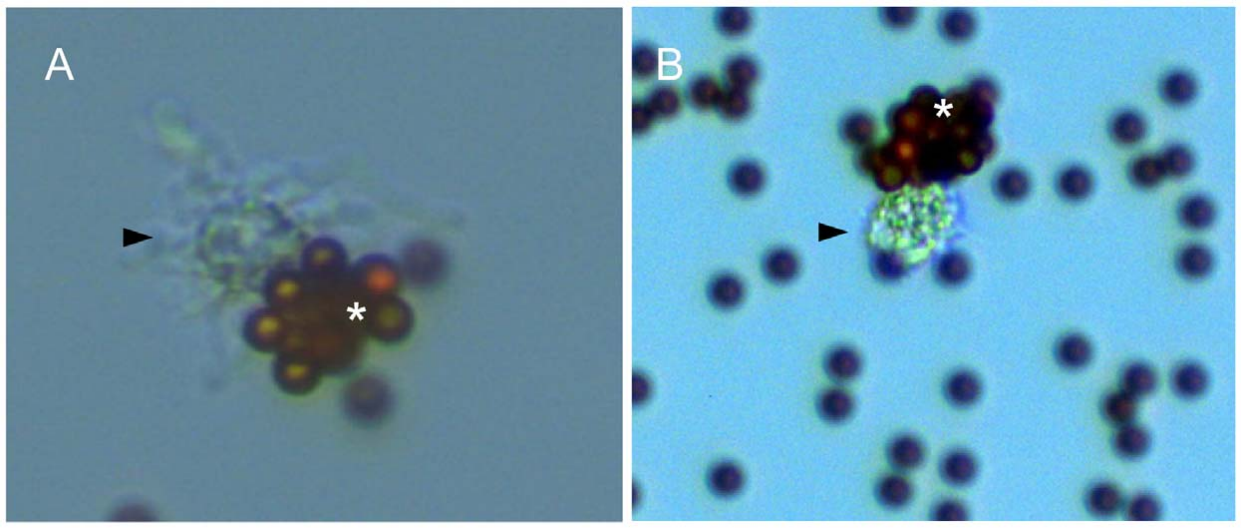
Different antigen presenting cells may present the same antigens to different T cells. Two lesional psoriatic T cells (*) labelled with CD8 beads are seen in direct contact with dendritic-like cells with antigen presenting cells of dendritic phenotype(▸, 200-fold magnification in an inverted microscope).

Direct microscopic analysis of the beads-labelled T cells allowed an interesting observation. In patient #4 each two CD8^+^ T cells from two clones (#2 and #3, Table 3) were seen in tight contact with target cells of either dendritic (DC) or epithelial-like morphology (Figure 3A, B). The contacts between the T cells and antigen presenting cell were so tight that they even survived the isolation procedure.

### TCR Analysis in Cryosections from Brain Lesions of Multiple Sclerosis

**Figure 4:**
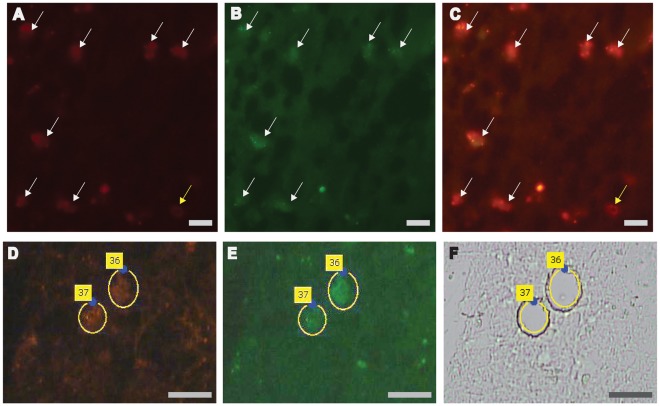
Localization of CD8^+^ T cells infiltrating the MS brain and their isolation by laser microdissection. Cryosections of a frozen biopsy sample from the MS patient were stained with Cy3-labeled anti-CD8β (red) and Alexa 488-labeled anti-CD134 (green) antibodies. (**A–C**): Visualization of activated T cells (white arrows) that are double positive (**C**) for CD8 (**A**) and CD134 (**B**). (**D–F**): Laser microdissection of T cells stained for CD8β (red; **D**) and CD134 (green; **E**). Activated single cells are dissected at the indicated yellow circles and catapulted out of the tissue directly into the cap of a PCR tube for subsequent TCR analysis. We show the corresponding bright-light image of the tissue after (**F**) laser microdissection. The numbers in the yellow field refer to apparatus parameters (**D–F**). The scale bars correspond to 20 µm.

In many conditions only frozen biopsy specimens are available. Furthermore, it may be of interest to precisely define the localisation of particular pathogenic T cells in tissue lesions by immunohistochemistry. Therefore, we tested whether our technology would be suited to analyze T cells from frozen biopsy samples using brain tissue sections from a patient with MS. We stained sections from three different tissue blocks with antibodies specific for the CD8 beta-chain and the T cell activation marker CD134 to ensure that we selectively isolated activated effector T cells, which are more likely to be the mediators of inflammatory demyelination (Figure 4).

We analysed the TCRs from 643 activated, i.e. double-stained CD8 beta^+^/CD134^+^ single effector T cells isolated by laser microdissection (Figure 4) from three different tissue blocks of one big lesion. In 68 of these formerly frozen and then immuno-stained T cells functional TCR β-chain rearrangements could be identified (yield: 10.6%) (Table 5, left panel). Of these, four TCR β-chain rearrangements were detected more than once. We found a Vβ8.1-chain 46 times (clone #8), a Vβ6.5-chain 8 times (clone #6) and a Vβ13.1-chain 3 times (clone #10) (Table 6). In 13 of the T cells we could also identify the paired α-chain (Table 5, right panel, Table 6). Of note, cells of the same T-cell clones were found not only in different tissue blocks, but were equally distributed in several morphologically distinct regions of the lesion (Table 6). This widespread clonal expansion points to the processing and presentation of identical or similar antigenic epitopes by antigen presenting cells in different parts of the brain. Accordingly, our technology may not only define clonal αβ TCRs *in situ* but also follow their precise localisation across pathologic T-cell infiltrates.

**Table 5:** TCR rearrangements of microdissected T cells from MS biopsy tissue.

			CDR3β							CDR3α			
Clone #	T cells identical/total	TCR Vβ-gene	Vβ	NDN	Jβ	Jβ-gene	Subclone	Frequency	TCR Vα-gene	Vα	NN	Jα	Jα-gene
1	1/68	6S2	CASS	PYPH	TEAFF	1.1			none				
2	1/68	6S2	CASS	SRDRG	GYTF	1.2			none				
3	1/68	6S2	CASSL	RPN	GELFF	2.2			none				
4	1/68	6S2	CASS	PTSL	TDTQYF	2.3			none				
5	1/68	6S3	CASSL	AFTGES	EQYF	2.7			none				
6	8/68	6S5	CASSL	APN	GELFF	2.2	6a	2/8	20S1	CLVGD	SRKG	DDKIIF	30
							6b	2/8	20S1	CLVG	AT	GNTGKLIF	37
7	1/68	8S1	CAS	THRGHG	NTEAFF	1.1			none				
8	46/68	8S1	CAS	TQGWGD	TEAFF	1.1	8a	1/46	1S5	CAV	SA	TDKLIF	34
							8b	7/46	30S1	CAV	PF	DRGSTLGRLYF	18
9	1/68	13S1	CASS	TSPGGARG	GNTIYF	1.3			none				
10 **¤**	3/68	13S1	CASS	LGA	DTQYF	2.3			none				
11 **¤**	1/68	13S2	CAS	RALVAT	YNEQFF	2.1			21S1	CAAS	G	GSNYKLTF	53
12	1/68	21S3	CASS	LAY	GELFF	2.2			none				

See legend to Table 2 for details; none: no TCR chain identified. **¤**: detected in earlier studies, see text. Subclones 6a,b and 8a,b: different α rearrangements identified with the same β rearrangement in different T cells.

**Table 6:** Distribution of T cells isolated by laser microdissection from morphologically distinct regions of the MS lesion.

	β-chains			
T cell clone #	Block #1	Block #2	Block #3	Total
1			1	1
2			1	1
3	1			1
4	1			1
5	1			1
6	5	3		8
7	1			1
8	23	13	10	46
9	1			1
10	1	2		3
11			1	1
12	1	1		2
13	1			1
**Total**	36	19	13	**68**
	**α-chains**			
6a	1	1		2
6b	1	1		2
8a	1			1
8b	5	1	1	7
11			1	1
**Total**	8	3	2	**13**

We list the numbers of T cells and tissue blocks where TCR β-chains (upper panel) and α-chains (lower panel) from particular T cell clones were detected.

Two clones, #10 and #11, had been detected as expanded clones in earlier studies by other techniques [17], [18]. According to homology searches using the protein query tool of Blast (http://blast.ncbi.nlm.nih.gov/Blast.cgi?PAGE=Proteins) all other PV and MS β chains represented unique rearrangements not identified before.

### Identification of T Cells with Dual TCRs

Several expanded clones expressed one β-chain in combination with two different α-chains. In PV, the T cells of two CD8^+^ T-cell clones of patient #3 (Vβ6S5 clone, Vβ6S3 clone), two CD8^+^ clones of patient #4 (clones #5, #10) and of the CD8^+^ clone #1 of patient #5 showed two different functional Vα rearrangements each (Tables 2, 3, 4).

The two strongly expanded MS TCR chains (Vβ8.1- and Vβ6.5-chains, MS clones #6 and #8) were each found in combination with two different α-chain rearrangements as well (Table 5), however, in different T cells. The Vβ6.5-chain of MS clone 6 was seen either in combination with a Vα20S1/Jα30 rearrangement (subclone 6a) or a Vα20S1/Jα37 rearrangement (subclone 6b). The Vβ8.1-chain of clone 8 was identified either with a Vα1S5/Jα34 (8a) or Vα30S1/Jα18 (8b) rearrangement. Dual TCRs may have two different specificities. This is of particular interest, because T cells with dual αβ-TCRs have been implicated in T-cell mediated autoimmunity [19], [20].

## Discussion

Here we describe a PCR-based technology which to our knowledge for the first time allows for an unbiased molecular analysis of matching i.e. paired TCR α- and β-chains from single human T cells. It employs a set of degenerate Vβ-gene primers and the introduction of an anchor primer site at the 5′ end of the PCR products of Vβ-gene rearrangements for further transcript amplification. Together with previously designed primer sets for the complete Vα repertoire [2] this allows us to characterize heterodimeric TCRs from single αβ T cells. The sensitivity of the method correlated with the method of isolation and accordingly mRNA preservation of the single T cells. It reached 80% for freshly isolated viable T cells aspirated by manual pipetting, but even pre-stained and laser-dissected T cells from frozen brain sections yielded an output sufficient to characterize the T cell infiltrates.

The technology has several obvious advantages. It is independent from the availability of monoclonal anti-Vβ region antibodies. Neither a prior knowledge of the TCR α- and β-chain regions nor preceding TCR spectratyping or other methods for the detection of expanded clones are required. This is documented by the analysis of the MS lesion: Two of the TCR rearrangements described here, #10 and #11 (Table 5), had already been identified formerly by single cell PCR using rearranged genomic DNA as PCR-template [17] or CDR3 spectratyping of the TCR β-chain repertoire [18]. In contrast to these earlier studies, however, we now could also characterize the corresponding α-chains, and we identified other clonal T cells from the same tissue blocks, which formerly had escaped detection.

T cell clones prevailed in skin lesions from psoriasis, as well. Similar to MS [10], [18], [21], [22] this had formerly been proposed from TCR β-chain repertoire studies [6], [7], [8], [9], [14]. We could now document that indeed numerous T cells of the psoriatic T-cell infiltrate carry identical αβ TCRs, and in accordance with the HLA-Cw6 association of PV [23] this clonality was particular evident for CD8^+^ T cells.

Identification of αβ-heterodimers by our technology provided definite evidence that completely identical clones may be present in morphologically distinct regions of human autoimmune tissue lesions. In MS considerable numbers of identical T cells were observed (Table 6). Such pervasive T cell clones [11] are obviously a quite general phenomenon in MS [10]. In psoriasis, the CD8^+^ T-cell clones predominated in the epidermis, indicating that the epidermis may be the actual site of psoriatic T-cell activation. And indeed, blocking T cell transmigration from the dermis into the epidermis by α1β1 integrin antibodies in a human-mouse skin transplant model may suppress psoriatic inflammation [16]. Identification of the clonal Vβ signature in blood and tonsils of a patient with streptococcal driven psoriasis is in line with our former observation that the pathogenic T cell clones are not primary skin-resident T cells but may be recruited from lymphoid organs such as the tonsils via the blood stream [9].

Thus, our technology facilitates the characterization of complex T-cell populations and helps to distinguish pathologically relevant T-cell clones from abundant bystander T cells of inflammatory infiltrates. It may therefore be used to characterize the distribution and expansion of particular T-cell clones across tissues or body fluids. Such T-cell clones may even be identified from low numbers of cells, where statistical techniques like CDR3 spectratyping [24] are not applicable. In PV less than 40 cells were sufficient to detect clonal T-cell expansions, as verified by analysis of an adjacent part of the biopsy.

αβ-TCR analysis may help to identify the respective antigen-presenting events. In PV different T cells with identical TCRs were seen in direct tight contact with cells of dendritic or epithelial morphology. So far direct evidence of an antigen-presenting event *in situ*, i.e. in the autoimmune tissue lesion, has been rare. The “*in flagranti*” observation of a likely antigen-presenting event in a psoriasis explant (Figure 4) now provides evidence that distinct antigen-presenting cells in psoriatic lesions may present a dominant antigen to different T cells of the same clonal origin.

The high sensitivity of our technology is reflected by its ability to detect dual TCRs on the single cell level. We identified several CD8^+^ T-cell clones from skin lesions of the PV patients #3, 4 and 5, which had two different α-chains associated with the same Vβ-chain rearrangement, respectively (Tables 2, 3, 4). Likewise, clones #6 and #8 from the MS lesion had two different α-chains associated with a Vβ6S5- or Vβ8S1-chain (Table 5). In the MS patient two possibilities are conceivable: Either the antigen-driven T-cell expansion selected two different T-cell clones each with the same TCR β-chain but different α-chains, or the T-cell clones expressed two different Vα-chains in a single dual TCR T cell, but only one α rearrangement was identified due to a low mRNA preservation. Dual-TCR T cells have been described, and it is assumed that up to 30% of all T cells may carry dual TCRs [19]. In case of the MS clones, we may not distinguish these possibilities, because the two α-chains were cloned from different cells with the same β-chain. However, the psoriasis clones were classical dual TCR T cells, as the two different α-chains were definitely cloned from the same T cells. This is the first time that clonal T cells with dual αβ-TCRs were identified directly from human autoimmune tissue lesions. These T cells likely have two different antigen-specificities, which may be relevant for the induction of autoimmunity. In a mouse model of autoimmune encephalitis employing a dual TCR specific for an exogenous viral antigen and myelin basic protein viral infection could brake tolerance to MBP and induce autoimmune pathology [20] hinting to such a mechanism in humans as well.

One of the greatest challenges in clinical immunology is the identification of antigens of T-cell mediated autoimmune disorders or tumor-specific immune responses. The technical hurdles, however, are high [1]. The molecular characterization of the paired αβ heterodimers as described here and the identification of T-cell clones on the single cell level clearly supports that the immune response in both disorders, PV and MS, is antigen-driven. Thus, our technology will now allow us to recombinantly reconstruct functional TCRs from practically any αβ-T cell of choice. Such “resurrected” T cells may then be used to identify the original antigen-specificity by a most recently developed generally applicable technology [25] or to test the functional outcome of T-cell mediated immune reactions in TCR transgenic animals. This application may significantly promote investigations on T-cell mediated autoimmune disorders, tumor-specific protective immunity, and infections.

## Materials and Methods

### Patients

The ethics committee of the medical faculty of the Ludwig-Maximilian-University of Munich had approved the study. Patients with type 1 chronic plaque psoriasis (early disease onset, positive family history for PV and/or presence of HLA-Cw6) [23] participated voluntarily and gave written informed consent. Clinical data of the MS patient are described [17], [18]. The biopsy was frozen in liquid nitrogen immediately upon excision.

### Isolation of Single T Cells from Peripheral Blood or Lesional Psoriatic Biopsies

Small spindle shaped biopsies from chronic PV plaques were cut in half. One part (“A”) was minced completely mechanically and cultured for 12 to 24 h in RPMI medium (PAA Laboratories) with standard supplements to allow T cells to segregate from the tissue threads. In some experiments, dermis and epidermis were dissociated by dispase treatment (2.4 U/ml, Roche) for 1 h at 37°C [26] and the T cells isolated from these two different compartments separately. Part “B” of the biopsy was homogenized in Trizol reagent for the preparation of mRNA and TCR β-chain cDNA. Peripheral blood lymphocytes (PBL) were prepared from heparinised venous blood by Ficoll density gradient centrifugation.

To isolate single CD4^+^ or CD8^+^ T cells, explant culture cells or PBL were suspended in PBS pH 7.2, 2 mM EDTA (autoMACS™ Rinsing Solution, Miltenyi Biotec), 0.5% bovine serum albumin (BSA) and incubated with anti-CD4 or anti-CD8 magnetic beads (Dynabeads, Invitrogen). Beads-coated cell suspensions were transferred into 6 well microtiter plates (Costar, Corning) and adjusted to a concentration of 1 to 2 beads-coated T cells per visual field at 200-fold magnification in an inverted microscope. Single beads-coated T cells were aspirated in a volume of ∼0.5 µl using a variable 2 µl pipette, transferred into PCR tubes containing 5.5 µl 1x OneStep RT-PCR buffer (Qiagen) and stored at −80°C. For FACSorting of single T cells, PBMC were stained with the anti-CD3 antibody UCHT1 (Dako), and single cell were sorted into PCR tubes using a FACSVantage SE cell sorter.

### Isolation of Single Cells from Frozen Brain Tissue by Laser Microdissection

PET membrane slides (P.A.L.M Microlaser) were baked at 180°C for 4 h, UV irradiated and coated with poly-L-lysine hydrobromide (Sigma). 10 µm thick cryostat sections from frozen tissue specimens were mounted onto these slides and stored at −80°C. Prior to staining, the slides were thawed, fixed briefly in 100% acetone rehydrated in PBS for 10 seconds, and blocked for 3 minutes in PBS containing 2% BSA (Sigma). Next, the sections were co-incubated for 5 minutes with an FITC labelled anti CD134 Antibody (clone ACT35, BD-Pharmingen) and a Cy3 labelled anti-CD8 beta chain antibody (clone 2ST8.547, Beckman Coulter). The sections were then rinsed with 1 ml of PBS and incubated for 3 minutes with a 1∶100 diluted Alexa-488 labelled anti-FITC antibody (A11096, Invitrogen) to enhance the fluorescence signal.

After a second PBS rinse the sections were covered with 1-propanol to retard RNase activity and prevent specimen drying. They were immediately analyzed under a P.A.L.M Microbeam-Z microscope (Zeiss). Cells that were either double positive for both T cell markers or single positive for CD8β were marked electronically using the PalmRobo Software (V3.0, Zeiss). After evaporation of 1-propanol, the cells were cut and catapulted by laser pressure into the mineral oil coated lids of single reaction 200 µl PCR tubes.

### Primer Design

All functional human TCR β-chain nucleotide sequences from the IMGT database at http://www.imgt.org/were searched for regions of nucleotide sequence homology using DNA sequence analysing software programs including “Geneious” (http://www.geneious.com); “CLC workbench” (http://www.clcbio.com); and “FastPCR” (http://www.biocenter.helsinki.fi/bi/Programs/fastpcr.htm). Melting temperatures and potential primer-primer interactions were determined using the IDT oligoanalyzer software (http://eu.idtdna.com/analyzer/applications/oligoanalyzer/default.aspx).

We use the TCR nomenclature of Arden et al. [27] throughout.

### Reverse Transcription and Pre-amplification of TCR α- and β-chains by Multiplex PCR

cDNA synthesis was performed directly from single T cells using the OneStep RT-PCR Kit. 6.5 µl of RT reaction mix with 10 µM of the Cα-*out* and Cβ-*out* oligonucleotide primers (Table 1) were added to the 6.0 µl sample containing the single T cell. Reverse transcription of TCR mRNA was performed for 35 min at 50°C. For cells isolated by laser microdissection, 12.5 µl of the RT mix were added directly into the cap of the reaction tube followed by centrifugation at 14,000 rpm, 4°C for 3 minutes.

After reverse transcription 12.5 µl PCR mix from the OneStep RT-PCR Kit were added to the sample. The PCR mix contained 3 µM Vα- and Vβ-oligonucleotide primers each. The primer set for the Vα-repertoire consisted of the 24 oligonucleotide primers described recently [2]. The primer set for the Vβ repertoire consisted of 9 oligonucleotide primers (Vp1 to Vp9) given in Table 1. After an initial incubation for 15 min at 95°C to activate hot start polymerase, PCR was run for 10 cycles at 94°C for 30 sec, 60°C for 90 sec and 68°C for 60 sec. Then another 30 cycles were run at 94°C for 30 sec, 53°C for 90 sec and 68°C for 60 sec, followed by a final extension at 68°C for 15 min. This pre-amplification product served as template for the subsequent characterization of TCR α- and β-chains.

### Characterization of the TCR β-chains

To facilitate a further amplification of the TCR β-chains by PCR, a universal primer (UP)-sequence was added as an anchor sequence to the 5′-end of each Vp1 to Vp9 primer (Vp1-UP to Vp9-UP). The nucleotide sequences of Vp2-UP and Vp9-UP were slightly modified to avoid primer interactions (Table 1). 1 µl of the multiplex PCR product was subjected to a run-off reaction in a PCR mix composed of 1 µl 10x PCR buffer (Roche), 0.2 µl dNTP (10 mM each), 7.65 µl H_2_O, 0.1 µl Vp1-UP to Vp9-UP primers (11.1 µM each), 0.05 µl Taq DNA Polymerase (5 U/µl, Roche). The run off conditions were 94°C for 5 min, 53°C for 150 sec, and 68°C for 15 min.

The TCR β-chain transcripts were then amplified by semi-nested PCR. 1 µl of the run-off reaction product was used in 20 µl PCR reaction mix that contained 2.5 µM of the Cβ-specific nested primer (Cβ-*in*) and 2.5 µM of the universal primer (UP) (Roche). After pre-incubation at 94°C for 2 min, PCR was run for 50 cycles at 94°C for 30 sec, 58°C for 1 min, 68°C for 1 min. After a final elongation step at 68°C for 15 min, PCR products were analyzed by agarose gel electrophoresis and sequenced.

### Characterization of the Matching TCR α-chains

Only such cells were tested, which had yielded a PCR product for the β-chain. The TCR α-chains were amplified by nested PCR from the pre-amplification product as described [2] with minor modifications: The concentrations of the Vα-*in* primers were raised to 1.0 µM, and the Cα-*in* primers were used at 5 µM. Further, the touch-down PCR was run with 4 cycles and 1 min each for the annealing temperatures 61°C, 58°C, and 56°C. Then 40 cycles were run at 53°C. Denaturation and extension steps were for 30 sec at 95°C and for 1 min at 68°C. PCR products were analyzed by agarose gel electrophoresis and sequenced.

### Analysis of the TCR α- and β-chain Repertoires from Blood T Cells

The Vα- and Vβ-TCR repertoires from PBMC were reverse transcribed and pre-amplified using the multiplex PCR conditions described above. Individual TCR Vα- and Vβ-gene families were then amplified from the multiplex PCR product in separate PCR reactions and analyzed by agarose gel electrophoresis or by CDR3 fragment lengths spectratyping as described [2], [14]. Only the primers for TRBV8 (5′ CCAGCCCTCAGAACCCAG 3′), TRBV21 (5′ CTCTCAGGATCCAGCCTGCA 3′) and TRBV25 (5′ GATTTTCAGCTAAGTGCCTCC 3′) differed from these protocols, because the original primers for TRBV8 and TRBV21 were located outside the multiplex β-chain PCR product, and the primer set did not include a primer for the TRBV25 family.

### Detection of Particular TCR Chains by Clone-specific PCR

For select clonal TCR rearrangements detected by single cell PCR in sample “A” of PV biopsies we investigated by clone-specific semi-nested RT-PCR whether their particular TCR β-chain transcripts were also present in cDNA of part “B”. The first PCR employed the respective Vβ forward primer and the Cβ-*out* primer. Then, in a semi-nested PCR, the Vβ forward primer and a CDR3 specific primer, which extended 4 to 6 nucleotides from the J-region gene into the respective N(D)N-sequence were used. PCR products were sequenced.
